# Computer Aided Diagnostic Support System for Skin Cancer: A Review of Techniques and Algorithms

**DOI:** 10.1155/2013/323268

**Published:** 2013-12-23

**Authors:** Ammara Masood, Adel Ali Al-Jumaily

**Affiliations:** School of Electrical, Mechanical and Mechatronic Engineering, University of Technology, Broadway Ultimo, Sydney, NSW 2007, Australia

## Abstract

Image-based computer aided diagnosis systems have significant potential for screening and early detection of malignant melanoma. We review the state of the art in these systems and examine current practices, problems, and prospects of image acquisition, pre-processing, segmentation, feature extraction and selection, and classification of dermoscopic images. This paper reports statistics and results from the most important implementations reported to date. We compared the performance of several classifiers specifically developed for skin lesion diagnosis and discussed the corresponding findings. Whenever available, indication of various conditions that affect the technique's performance is reported. We suggest a framework for comparative assessment of skin cancer diagnostic models and review the results based on these models. The deficiencies in some of the existing studies are highlighted and suggestions for future research are provided.

## 1. Introduction

The incidence of melanoma skin cancer has been increasing over the past few decades [1–3]. Estimated 76,250 new cases of invasive melanoma were diagnosed in USA in 2012, with an estimated number of 9,180 that result in death [4]. Australia has one of the highest rates of skin cancer in the world. Over 1,890 Australians die from skin cancer each year [5]. Melanoma is capable of deep invasion. The most dangerous characteristic of melanoma is that it can spread widely over the body via the lymphatic vessels and blood vessels. Thus, early diagnosis of melanoma is a key factor for the prognosis of the disease.

The usual clinical practice of melanoma diagnosis is a visual inspection by the dermatologist. Clinical diagnostic accuracy is a bit disappointing [6, 7]. However, dermoscopy [8] is a noninvasive diagnostic technique that links clinical dermatology and dermatopathology by enabling the visualization of morphological features which are not discernible by examination with the naked eye. There are different techniques, like solar scan [9], epiluminescence microscopy (ELM) [10, 11], cross-polarization epiluminescence (XLM), and side transillumination (TLM) [12, 13], that can greatly increase the morphological details that are visualized. Thus, they provide additional diagnostic criteria to the dermatologist.

Dermoscopy enables better diagnosis as compared to unaided eye [14–16] with an improvement in diagnostic sensitivity of 10–30% [17]. However, it has also been demonstrated that dermoscopy may actually lower the diagnostic accuracy in the hands of inexperienced dermatologists [10, 18–20], since this method requires great deal of experience to differentiate skin lesions [21]. As described in [9, 22] only experts have arrived at 90% sensitivity and 59% specificity in skin lesion diagnosis, while for less trained doctors these figures show significant drop till around 62%-63% for general practitioners.

The main problem is that the diagnosis is highly dependent on subjective judgement and is scarcely reproducible [23, 24]. Several scoring systems and algorithms such as the ABCD-E rule [25–27], the seven-point checklist [28–30], three-point checklist [31], and the Menzies method [32, 33] have been proposed to improve the diagnostic performance of less experienced clinicians. Although this simplification has enabled the development of these diagnostic algorithms with good accuracy, still they showed problems that have not yet been solved. The most important shortcoming is that the purpose for which they were designed was not achieved, because the within- and between-observer concordance is very low, even for expert observers [10, 25, 34, 35]. Despite extensive research in investigating the varied presentations and physical characteristics of melanoma, the clinical diagnostic accuracy remains suboptimal. Thus, a growing interest has developed in the last two decades in the automated analysis of digitized images obtained by ELM techniques to assist clinicians in differentiating early melanoma from benign skin lesions.

Application of computational intelligence methods helps physicians as well as dermatologists in faster data process to give better and more reliable diagnoses. Studies related to the automated classification of pigmented skin lesion images have appeared in the literature as early as 1987 [36]. After some successful experiments on automatic diagnostic systems for melanoma diagnosis [36–42], utility of machine vision and computerized analysis is getting more important every year. The importance of the topic is patent if we analyse the enormous quantity of research works related with the melanoma diagnosis. Numerous computerized diagnostic systems have been reported in the literature where different border detection, feature extraction, selection, and classification algorithms are used. Some researchers [37, 43–48] reviewed and tried to critically examine image analysis techniques for diagnosis of skin cancer and compared diagnostic accuracy of experts dermoscopists with artificial intelligence and computer aided diagnosis. More research, however, is needed to identify and reduce uncertainties in the automatic decision support systems to improve diagnosis accuracy. A comprehensive up-to-date review of automatic diagnostic model for skin lesions is not available. Continuous emergence of new classification algorithms and techniques for dermoscopic image analysis in recent years necessitates such a review.

This paper describes a standard automatic decision support system which is based on semantic analysis of melanoma images and further classification of characteristic objects commonly found in pigmented skin lesions. The aim of this review is to summarize and compare advanced dermoscopic algorithms used for the classification of skin lesions and discuss important issues affecting the success of classification. A brief and comprehensive review of feature extraction and selection algorithms that are so far being used for extracting various features of malignant melanoma is also provided. Analysis of various papers is performed with respect to several criteria, such as lesion segmentation, feature extraction, size of data sets, classification techniques, and performance measures used in reporting the diagnosis results. This paper will provide a framework that represents a comprehensive guideline for selecting suitable algorithms needed for different steps of automatic diagnostic procedure for ensuring timely diagnosis of skin cancer.

The paper is organized as follows. the scheme of a general computer aided diagnosis system is provided. A comprehensive review of the available literature regarding each stage is presented. The different classification algorithms are explained. Performance evaluation measures and model validation details are presented for analysing various algorithms/models and finally concluding comments are provided.

## 2. Computer-Aided Diagnosis System

Computer aided decision support tools are important in medical imaging for diagnosis and evaluation. Predictive models are used in a variety of medical domains for diagnostic and prognostic tasks. These models are built based on experience which constitutes data acquired from actual cases. The data can be preprocessed and expressed in a set of rules, such as that it is often the case in knowledge-based expert systems, and consequently can serve as training data for statistical and machine learning models.

**Figure 1:**
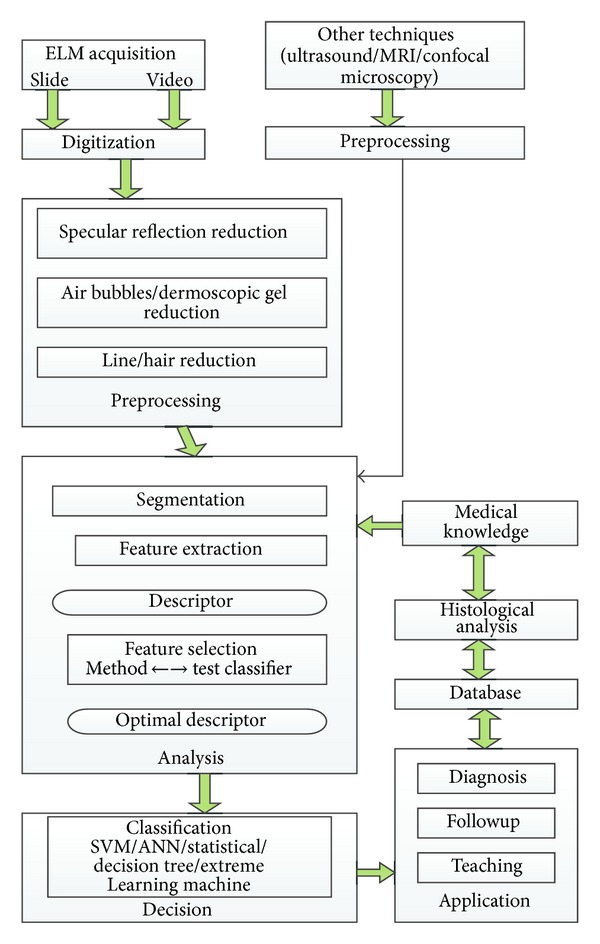
Computer aided diagnostic support system for skin cancer diagnosis.

The general approach of developing a CAD system for the diagnosis of skin cancer is to find the location of a lesion and also to determine an estimate of the probability of a disease. The first step in this paper was to establish a standard general scheme of a CAD system for skin lesions. The proposed scheme is shown in Figure 1. The inputs to the computer aided system are digital images obtained by ELM, with the possibility to add other acquisition system such as ultrasound or confocal microscopy. In the first phase preprocessing of image is done that allows reducing the ill effects and various artifacts like hair that may be present in the dermoscopic images. It is followed by the detection of the lesion by image segmentation technique. Once the lesion is localized, different chromatic and morphological features can be quantified and used for classification.

Differentiation of malignant melanoma images demands very fast image processing and feature extraction and classification algorithms. A detailed research is necessary to make the best choice and to set the benchmarks for diagnostic system development and validation. The following section focuses on the description of the major steps that may be involved in skin cancer diagnosis.

### 2.1. Image Acquisition/Methods for Screening Skin Lesions

Unaided visual inspection of the skin is often suboptimal for diagnosing melanoma. Numerous imaging modalities are under investigation to determine their usefulness in imaging and ascertaining a correct in vivo diagnosis of melanoma. These include total cutaneous photography, dermoscopy, confocal scanning laser microscopy (CSLM), ultrasound, magnetic resonance imaging (MRI), optical coherence tomography (OCT), and multispectral imaging. Each technique has certain pros and cons. These are now being harnessed to improve early detection. We have provided here a bird eye view of the currently available cutaneous imaging devices and new frontiers in noninvasive automated diagnosis of melanoma in Table 1. Readers may refer to [33, 49–52] for analysing performance comparison of some of the existing screening techniques.

Relative to other specialties, dermatologists have been slow to adopt advanced technologic diagnostic aids. Thus, so far dermoscopy is the fastest growing method to image skin. Sometimes simple ELM does not sufficiently increase the diagnostic accuracy in distinguishing pigmented Spitz nevus (PSNs) from melanoma. For obviating the problems of qualitative interpretation, methods based on the mathematical analysis of pigmented skin lesions (PSLs), such as digital dermoscopy analysis (DDA) and D-ELM, have been developed [53, 54]. The visual evaluation of the content of DDA is very complex. Efficient image processing techniques must therefore be developed to help physicians in making a diagnosis. The introduction of digital ELM and sophisticated image processing software has opened up a new horizon in the evaluation of cutaneous benign and malignant pigmented skin lesions (PSLs) as it enables the observation, storage, and objective evaluation of many parameters.

In this paper we have focussed on automatic diagnostic system based on digital dermoscopy images normally collected from different dermoscopy atlases [55, 56] or from dermatologists since it is the most widely used. However, we anticipate that multimodal systems that combine different imaging technologies will further improve the ability to detect melanoma at an earlier stage and reduce the trauma of dermatologic diagnosis.

### 2.2. Preprocessing

The main processing step towards a complete analysis of pigmented skin lesion is to differentiate the lesion from the healthy skin. Detection of the lesion is a difficult problem in dermatoscopic images as the transition between the lesion and the surrounding skin is smooth and even for trained dermatologist; it is a challenge to distinguish accurately. It has been observed that dermoscopy images often contain artifacts such as uneven illumination, dermoscopic gel, black frames, ink markings, rulers, air bubbles, and intrinsic cutaneous features that can affect border detection such as blood vessels, hairs, and skin lines and texture. These artifacts and extraneous elements complicate the border detection procedure, which results in a loss of accuracy as well as an increase in computational time. Thus, it requires some preprocessing steps to facilitate the segmentation process by the removal of unwanted objects or artifacts and colour space transformation.

Everything that might corrupt the image and consequently affect the results of image processing must be localized and then removed, masked, or replaced. Many approaches can be used that include image resizing, masking, cropping, hair removal (or attenuation), and conversion from RGB color to intensity grey image. It is done to reduce noise and the effect of reflection artifacts. It is meant to facilitate image segmentation by filtering the image and enhancing its important features.

The most straightforward way to remove these artifacts is to smooth the image using a general purpose filter such peer group filter (PGF) [57], mean filters, median filter [58–60], Gaussian filters [61, 62], or anisotropic diffusion filters (ADF). A major issue with these aforementioned filters is that these filters are originally formulated for scalar images. For vector images one can apply a scalar filter to each channel independently and then combine the results, a strategy referred to as marginal filtering. Despite being fast, this scheme introduces color artifacts in the output. An alternative solution is to use filters that treat the pixels as vectors [63]. Another noteworthy thing is setting mask size proportional to the image size to manage a tradeoff between smoothing of image and blurring of edges. Inspite of taking care of all the forementioned things, it is still not guaranteed to get an image free of all artifacts.

An alternative strategy for artifact removal is to use specialized methods for each artifact type. Many methods have been suggested; very few [64–66] discussed different aspects of artifacts together, but none of them have discussed all cases of artifacts. For this rationale, we have presented an overview of effective preprocessing methods, namely, color space transformation, color quantization, contrast enhancement, and artifact removal, which are being used for reducing all the possible ill effects present in the dermoscopic images.

Dermoscopy images are commonly acquired using a digital camera with a dermatoscope attachment. Due to the computational simplicity and convenience of scalar (single channel) processing, the resulting RGB (red-green-blue) color image is often converted to a scalar image using different methods like retaining only the blue channel as lesions are often more prominent in this channel or applying the luminance transformation or Karhunen-Loéve (KL) transformation and retaining the channel with the highest variance. Skin lesions come in a variety of colors but absolute colors are not very useful in segmenting images. Normally the analysis is based on changes in color within the lesion or with the surrounding skin particularly color changes belonging to the lesion boundary. Therefore, it is quite common to transform the images that are in RGB color coordinates into other color spaces like CIEL*a*b*, CIEL*u*v*, KL, and HSI (Hue-Saturation-Intensity).

Typical 24-bit color images have thousands of colors, which are difficult to handle directly. For this reason color quantization is commonly used as a preprocessing step for color image segmentation [67]. The process of color quantization consists of two-phases palette design (i.e., selection of a small set of colors that represents the original image colors) and pixel mapping (i.e., assignment of one of the palette colors to each input pixel). Celebi et al. [57] showed that, for skin lesion, the color quantization method should reduce the number of colors in image to 20 for getting precise quantization.

One of the factors that complicate the detection of borders in dermoscopy images is insufficient contrast. The contrast of image is enhanced to ensure that edges of the lesion are eminence. Gómez et al. [68] proposed a contrast enhancement method based on independent histogram pursuit (IHP). An easy, yet powerful way to enhance the image contrast is histogram stretching, a mapping of the pixel values onto [0,255]. Another very popular technique is histogram equalization, which alters pixel values to achieve a uniform distribution. Homomorphic filtering [69], FFT, and high pass filter can be used to compensate for uneven illumination or specular reflection variations in order to obtain the high contrast lesion images.

For the removal of black frames produced in the digitization process, Celebi et al. [59, 70] proposed an iterative algorithm based on the lightness component of the HSL (Hue-Saturation-Lightness) color space. In order to remove air bubbles and dermoscopic gels, adaptive and recursive weighted median filter developed by Dehghani Tafti and Mirsadeghi [71] can be utilized. This type of median filters has an edge persevering capability. A method that can remove bubbles with bright edges was introduced in [72] where the authors utilized a morphological top-hat operator followed by a radial search procedure. Line detection procedure based on the 2D derivatives of Gaussian (DOG) [73] and exemplar-based object removal algorithm [74] can be used for removing dark lines like ruler marking. In most cases, image smoothing effectively removes the skin lines and blood vessels.

One of the most undesirable elements that are most commonly present in dermatoscopic images is hair. Lee et al. [75] and Schmid [76] used mathematical morphology. Fleming et al. [72] applied curvilinear structure detection with various constraints followed by gap filling. Erosion/dilation with straight line segments can efficiently eliminate (or at least weaken the effect of) hairs [77, 78]. Schmid et al. [79, 80] suggested a scheme based on a morphological closing operator, while in [81] they applied to the three components of the L*u*v* uniform color space [82]. Zhou et al. [83] and Wighton et al. [84]. proposed more sophisticated approaches based on inpainting. However, it is being observed that most of these techniques often leave behind undesirable blurring; disturb the texture of the tumor; and result in color bleeding. Due to these problems, it is very difficult to use the color diffuse image for further skin tumor differentiation. In contrast, a new artifact removal algorithm that focuses on accurate detection of curvilinear artifacts and pays special attention to lesion structure during the removal stage has been introduced by Zhou et al. [85]. This approach effectively removes artifacts such as ruler markings and hair, but it has high computational requirements.

To address all these issues Abbas et al. [64] developed a novel method that automatically detects these visible artifacts and removes them. Abbas et al. [86] presented a comparative study about hair removal methods which indicate that hair-repairing algorithm based on the fast marching method achieves an accurate result.

All the above mentioned strategies are meant to facilitate the segmentation and feature extraction stages which consequently lead to better diagnostic results.

### 2.3. Segmentation

Segmentation refers to the partitioning of an image into disjoint regions that are homogeneous with respect to a chosen property such as luminance, color, and texture. The goal of segmentation is to simplify and/or change the representation of an image into something that is more meaningful and easier to analyse. Some researchers [87] argued that manual border detection is better than computer-detected borders in order to separate the problems of feature extraction from the problems of automated lesion border detection. However, for the development of automated diagnostic system for skin lesion detection, it is very important to develop automatic segmentation algorithms. As segmentation is a crucial early step in the analysis of lesion images, it has become one of the important areas of research and many algorithms and segmentation techniques are available in the literature. We have briefly provided an overview of various segmentation algorithms being used for dermoscopic image analysis as tabulated in Table 2.

Several comparative studies [59, 61, 66, 88, 89] are also present in the literature which provides performance analysis of several segmentation algorithms. There are several issues that should be kept in mind for selecting a suitable algorithm, for example, scalar versus vector processing, automatic versus semiautomatic, and the number of parameters whose values need to be determined a priori [65]. Interested readers may check relevant references to identify a suitable approach for a specific study.

### 2.4. Feature Extraction

Melanoma is visually difficult to differentiate from Clark nevus lesions which are benign. It is important to identify the most effective features to extract from melanoma, melanoma in situ and Clark nevus lesions, and to find the most effective pattern-classification criteria and algorithms for differentiating those lesions. Thus, the next stage of the image analysis process is to extract the important features of the image.

The purpose of feature extraction is to reduce the original data set by measuring certain properties, or features, that differentiate one input pattern from another. The feature extraction is performed by measurements on the pixels that represent a segmented object allowing various features to be computed. Unfortunately, the feature extraction step is often subject to error. In most of the publications dealing with this topic, many features are extracted to feed a sophisticated classifier, but there is very little discussion about the real meaning of those features and about objective ways to measure them. Thus, we investigate this topic in detail to come up with a guideline for future research.

Different feature extraction methods found in the literature include statistical and model-based and filtering-based methods, among which multichannel filtering is the most efficient and accurate one. Various researchers used principal component analysis (PCA) of a binary mask of the lesion, wavelet packet transform (WPT) [90–94], grey level cooccurrence matrix (GLCM) [61, 95], Fourier power spectrum [96], Gaussian derivative kernels [97], and decision boundary feature extraction [98–100] in order to reduce data redundancy. Some of the typically used filter banks are Laws masks, the dyadic Gabor filter bank, and wavelet transform [101]. A particular problem in the related literature is that a significant number of studies do not report the details of their feature extraction procedure; see Table 6.

The ABCD-E system [25, 26, 102], 7-point checklist [29, 103], 3-point checklist [104], pattern analysis [23], and Menzies method [105] offer alternative approaches in deciding the differentiating features that need to be extracted.

According to the conclusion made by Johr [28], the automatic extraction of characteristics that take into account the rule ABCD [25, 102, 106] is computationally less expensive than the ones that take into account 7-point checklist [29, 103] or the Menzies method [32, 107]. Furthermore, the reliability in the clinical diagnosis is very high for ABCD-E rule. So, most of the automated decision support systems also use ABCD rule as the base of their feature extraction step. However, ABCD is more prone to over classification of atypical melanocytic nevi as melanomas. Dolianitis et al. [108] showed, in a comparative study, that Menzies method achieved the highest sensitivity, 84.6%, for the diagnosis of melanoma, followed by the 7-point checklist (81.4%), the ABCD rule (77.5%), pattern analysis (68.4%), and assessment of a macroscopic image (60.9%). Pattern analysis and assessment of the macroscopic image showed the highest specificity, 85.3% and 85.4%, respectively. So many researchers [109–114] are trying to develop efficient automatic diagnostic systems based on 7-point criteria and pattern analysis.

**Figure 2:**
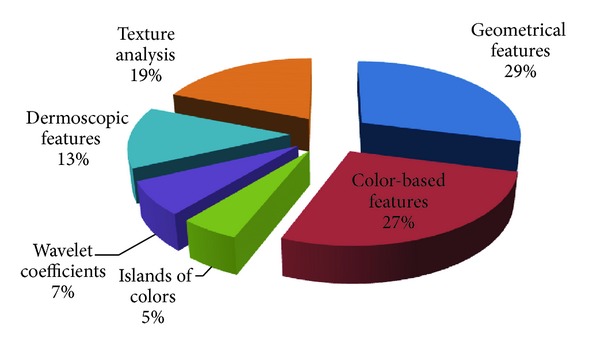
Illustration of feature distribution used in dermoscopic studies in the literature.

Numerous methods for extracting features from clinical skin lesion images have been proposed in the literature as Figure 2 illustrated the distribution of features used in dermoscopic studies. Several studies have also proven the efficiency of border shape descriptors for the detection of malignant melanoma on both clinical- and computer-based evaluation methods [115, 116]. Very simple parameters, such as area and perimeter, are extracted in [117–119]. Measurements of shape features are also used like fragmentation index [120–122], thinness ratio/circularity factor [61, 123–125] asymmetry index [77, 116, 122], aspect ratio [118, 126], compactness [118, 126], symmetry axis [127], bulkiness score [128], irregularity index [129, 130], fractality of borders [117], convex hull ratio [124], and skin line pattern [131]. Some groups use the sharpness of the transition from the lesion interior to the skin [61, 123, 125] as descriptors of the structure and irregularity of the border. Hall et al. [37] calculate fractal dimensions to represent border irregularity. Lacunarity [132] is another measure that can be used to characterize a property of fractals and quantifies aspects of patterns that exhibit changes in structure.

Color features are mainly statistical parameters calculated from different colour channels, like average value and standard deviation of the RGB [120–124] or HSV colour channels [125]. Other color features used in different studies include colour asymmetry [118], centroidal distance [118], and LUV histogram distance [118]. Cotton and Claridge [133] found that all normal skin colours lie on a two-dimensional surface patch within a three- dimensional (3D) colour space (CIE-LMS). Atypical skin structures result in colour coordinates that deviate from the normal surface patch. Some researchers [61, 117, 118, 134, 135] used GLCM-based texture features [136–138] like dissimilarity, contrast, energy, maximum probability, correlation, entropy, and so forth.

Parameters for the description of dermatoscopic structures and ELM criteria are difficult to find in the literature. Major issues are concerned with the difficulty in relating such information as lesion shape and color to medical structures (tissues, vessels, etc.) which experts are more familiar with. Some of the dermoscopic feature extraction studies include atypical pigment networks [72, 110, 139], globules/dots/blotches [72, 140–143], streaks [144], granularity [145], and blue-white veil [87, 146]. It is noteworthy that diagnostic systems based on extraction of critical high level features show an increase in the diagnostic accuracy of computerized dermoscopy image analysis systems. Thus, in addition to general features like area, border, shape, and color, these high level features should also be integrated in the automated diagnostic system to gain greater clinical acceptance.

Some researchers used some unique features for classification, but we know from skin cancer research that a unique feature is not sufficient to diagnose precisely skin cancer and that the combination of different criteria is the key to the early detection of malignant melanoma and other types of skin cancer. The evolution of competing dermoscopic algorithms with variable definitions of specific attributes complicates dermoscopic diagnosis. It is necessary to identify features that are the most reproducible and diagnostically significant and formulate them into a single algorithm.

### 2.5. Feature Selection

For clinical purposes, it is arguable that parsimony is a desirable feature of a good predictive model [147]. Similarly, features selection is a critical step for successfully distinguishing between malignant melanoma, benign, and dysplastic nevi. Many potential features may be used, but it is important to select a reasonable reduced number of useful features while eliminating redundant, irrelevant, or noisy features. However, it is important to make sure that there may not be loss of significant information.

From the classification perspective, there are numerous potential benefits associated with feature selection: (i) reduced feature extraction time and storage requirements, (ii) reduced classifier complexity for better generalization behaviour, (iii) increased prediction accuracy, (iv) reduced training and testing times, and (v) enhanced data understanding and visualization.

There are many methods available for feature selection [148] which include principle component analysis [81] and search strategies like sequential forward selection (SFS) [149], sequential backward selection (SBS) [150], plus-l-take-away-r (PTA (l, r)), floating search methods [54, 151], sequential forward floating selection (SFFS), sequential backward floating selection (SBFS)) and Fisher score ranking [135]. All these algorithms use stepwise inclusions and exclusions of features into/from the subset of consideration, but they differ in their strategy of applying them. Although the floating methods are considered to be more intelligent, they are still suboptimal and even more there is no warranty that they yield better results.

In addition to these, some of the filter-based methods include ReliefF [152], mutual information-based feature selection (MIFS) [153], and correlation-based feature selection (CFS) [154]. Filter methods are usually very fast and allow one to compare several alternative methods within an optimization framework. It is possible, and also desirable, to use clinical criteria or statistical methods to reduce the number of candidate variables, thus reducing the risk of an overoptimistic model [155].

A particular problem in the related literature is that there is very little number of studies that report the details of their feature selection procedure. Normally we do not find details of feature selection procedures that are used for choosing the appropriate features for skin cancer diagnosis. Handels et al. [156] described feature selection as an optimization problem and compared several approaches including heuristic strategies, greedy and genetic algorithms. Zagrouba and Barhoumi [157] proposed an accelerated system for melanoma diagnosis based on subset feature selection.

The number of features retained by the feature selection algorithm (*k*) is an important parameter. Sometimes a small number of features are not likely to discriminate between the classes well. On the other hand, a large number of features might lead to overfitting. Green et al. [121] showed by calculating correlation coefficients that the size of the lesion is the most important feature in their system. Roß et al. [158] perform a feature selection by the application of the sequential forward selection algorithm. They achieve a tremendous reduction to five features starting with 87 features calculated from surface profiles of skin lesions. Ganster et al. [159] used SBFS and SFFS and showed that the best selection performances were with subset size of between 10 to 15 and performance degrades with subsets size of more than 20 features. On the other hand by inspecting individual sensitivities on the malignant class of several subset sizes, it turns out that an acceptable performance is only achieved with subsets of more than 20 features. While Celebi et al. [118] showed by using CFS feature selection algorithm that AUC peaks can be obtained with the use of 18 features and inclusion of features beyond this value does not add much to the classifier performance.

Rohrer et al. [160] presented a study particularly based on feature selection for melanoma recognition and showed a strong increase in performance for small subsets followed by a slight increase up to medium sized subsets. Larger subsets cause a drop in the recognition rate. Ruiz et al. [126] also confirmed this thing in the evaluation done using SBFS and SFFS and showed that minimum error rate was observed using subset of 6 features and a significant increase in classification error rate is observed by using a subset of more than 20 features.

By inspecting the overall achieved performances one even could imagine that using 5 to 20 features is enough to get acceptable classification results. The aim of feature selection is to find the optimum number of features to obtain the best achievable performance (i.e., recognition rate) in classification. Therefore, the feature selection algorithms should be evaluated to get performance estimation on some standard classifier by applying tenfold cross-validation (XVAL), that is, repeating feature selection ten times with slightly different data for all algorithms.

### 2.6. Classification

Classification phase of the diagnostic system is the one in charge of making the inferences about the extracted information in the previous phases in order to be able to produce a diagnostic about the input image. There are two different approaches for the classification of dermoscopic images: the first considers only a dichotomous distinction between the two classes (melanoma and benign) and assigns class labels 0 or 1 to data item. The second attempts to model *P*(*y* | *x*); this yields not only a class label for a data item, but also a probability of class membership. The most prominent representatives of the first approach are support vector machines. Logistic regression, artificial neural networks, *k*-nearest neighbours, and decision trees are all members of the second approach, although they vary considerably in building an approximation to *P*(*y* | *x*) from data.

We do not intend in this paper to delve deeply into the technical aspects of all the classification algorithms. However, to make the reader analyse the performance of algorithms that are mostly used for dermoscopic image analysis, we believe that it is helpful to air them briefly. Readers who wish to have a detailed description of a specific classification approach should refer to cited references.

#### 2.6.1. *K*-Nearest Neighbour Algorithm

The *K*-nearest neighbour classifier [161, 162] is a nonparametric method of pattern recognition. For a lesion belonging to the test set (query vector), it is found that the *K* vectors are the closest to the query vector in the training set. The unclassified sample is then assigned to the class represented by the majority of the *K* closest neighbours.

The most critical requirement of the *K*-nearest neighbour classifier is to have a training set including enough examples of each class of pigmented lesions to adequately represent the full range of measurements that can be expected from each class. Optimizing the procedures of feature selection and weight definition could additionally improve the performance of the *K*-nearest neighbour classifier [163].

In medicine, most applications use nearest-neighbour algorithms as benchmarks for other machine learning techniques [156, 164]. Classification based on the *k*-nearest neighbour algorithm differs from the other methods considered here, as this algorithm uses the data directly for classification, without building a model first [162, 165]. The only adjustable parameter in the model is *k*, the number of nearest neighbours to include in the estimate of class membership, and the value of *P*(*y* | *x*) is calculated simply as the ratio of members of class *y* among the *k*-nearest neighbours of *x*. By varying *k*, the model can be made more or less flexible (small or large values of *k*, resp.). Generally, the choice of *k* can only be determined empirically.

*K*-NN algorithm permits retrieval and visualization of the “most similar” cases to those at hand. This aspect partly resembles the medical reasoning and allows a dermatologist to directly compare unknown lesions with other known skin lesions. This case-based explanation can provide an advantage in areas where black-box models are inadequate. It is well known that *k*-NN fails in case of irrelevant features. *K*-NN can also be used for the evaluation of feature subset selection process because it allows incorporating/eliminating characteristics easily and it has low computational cost.

The major drawback of *k*-nearest neighbour lies in the calculation of the case neighbourhood. Thus, it needs to define a metric that measures the distance between data items. In most application areas, it is not clear how to, other than by trial and error, define a metric in such a way that the relative (but unknown!) importance of data components is reflected in the metric [166].

#### 2.6.2. Decision Trees

The decision tree approach belongs to the supervised machine learning techniques. It is popular for its simplicity in constructing, efficient use in decision making, and simple representation, which is easily understood by humans.

This algorithm repeatedly splits the data set according to a criterion that maximizes the separation of the data, resulting in a tree-like structure [167–171]. It does this by identifying a variable and a threshold in the domain of this variable that can be used to divide the data set into two groups. The best choice of variable and threshold is the one that minimizes the disparity measures in the resulting groups. The most common criterion employed is information gain; this means that at each split, the decrease in entropy due to this split is maximized. The estimate of *P*(*y* | *x*) is the ratio of *y* class elements over all elements of the leaf node that contains data item *x*. Various modifications of decision trees like ADWAT and LMT are also used for dermoscopic image classification.

Advantages and disadvantages of decision trees in medicine have been widely investigated [172, 173]. The advantage of decision trees over many of the other methods is that they are not black-box models but can easily be expressed as rules. This makes them especially well-suited for medical applications. In many classification tasks decision tree classifiers have been preferred to other solutions (also including ANN and SVM) because they are often fast to train and apply and generate easy-to-understand rules.

A major disadvantage of decision trees is given by the greedy construction process. In this process at each step, the combination of single best variable and optimal split-point is selected. However, on the other hand if we use multistep look ahead, it considers combinations of variables which may obtain different (and better) results. Given a large training set, decision tree classifiers, in general, generate complex decision rules that perform well on the training data but do not generalize well to unseen data [174]. In such cases, the classifier model is said to have overfit the training data. A further drawback lies in the fact that continuous variables are implicitly discretised by the splitting process, losing information along the way.

#### 2.6.3. Logistic Regression

Logistic regression is an algorithm that constructs a separating hyperplane between two data sets, using the logistic function to express distance from the hyperplane as a probability of class membership.

Although the model is linear in parameters and can thus only calculate linear decision boundaries, it is nevertheless a widely used predictive model in medical applications [155, 175, 176]. The main advantage that this method has over other algorithms is its ease of use (it is implemented in numerous software packages), allowing the interpretation of results as probabilities and variable-selection capability. Dreiseitl et al. [166] showed in a comparative study that logistic regression performs on about the same level as artificial neural networks and support vector machines, which are both capable of implementing nonlinear separating surfaces.

#### 2.6.4. ANN

Artificial neural network [165, 177–180] is one of the great vital parts of soft computing. The ANN consists of several small processing units (the artificial neurons) that are highly interconnected. Information flow in an ANN is modelled after the human brain. The supervise ANN is an iterative process which requires many presentations of the training set; the system is said to learn from examples. It has conspicuous capacity to obtain idea from complex data and is used to take out patterns and determine trends that are too difficult to be noticed by humans or any other computer skills. A lot of research is being carried out nowadays on dermoscopic image analysis using ANNs.

**Figure 3:**
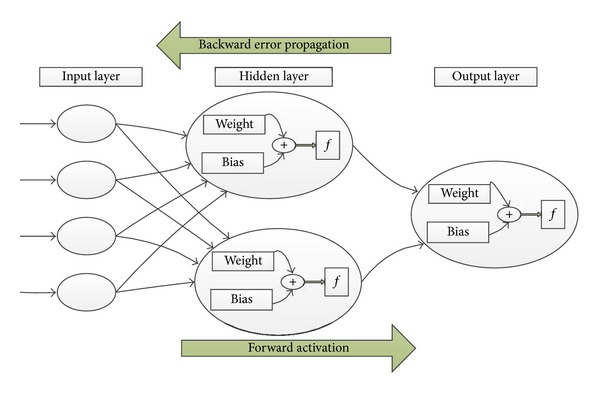
Working mechanism of artificial neural network.

The general working mechanism for artificial neural network is presented in Figure 3. Many of the early implementations required a significant amount of parameter tuning to achieve satisfactory results, a process that needed too much time and expertise for a nonexpert. Over the past few years, statistically motivated Bayesian methods [181] and implementations of faster learning algorithms [182] have allowed nonexperts use to sophisticated methods that require little to no parameter tuning. Various neural networks-based clustering techniques and algorithms are being used in this regard [183] which include back propagation network (BPN), radial basis function network (RBF) and extreme learning machine (ELM).

#### 2.6.5. Support Vector Machines

**Figure 4:**
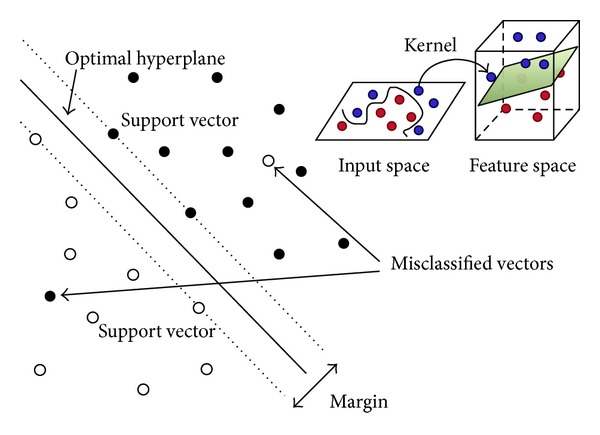
Principle of support vector machine.

Support vector machines (SVMs) are a machine learning paradigm based on statistical learning theory [184, 185]. Performances on par with or exceeding that of other machine learning algorithms have been reported in the medical literature. Algorithmically, support vector machines build optimal separating boundaries between data sets by solving a constrained quadratic optimization problem [186]. While the basic training algorithm can only construct linear separators, different kernel functions (i.e., linear, polynomial, radial basis function, and sigmoid) can be used to include varying degrees of nonlinearity and flexibility in the model. The principle of support vector machine is shown in Figure 4.

SVMs have several advantages over the more classical classifiers such as decision trees and neural networks. The support vector training mainly involves optimization of a convex cost function. Therefore, there is no risk of getting stuck at local minima as in the case of back propagation neural networks. SVMs are based on the structural risk minimization (SRM) principle which minimizes the upper bound on the generalization error. Therefore, SVMs are less prone to overfitting when compared to algorithms such as back propagation neural networks that implement the ERM empirical risk minimization principle. Another advantage of SVMs is that they provide a unified framework in which different learning machine architectures (e.g., RBF networks and feed forward neural networks) can be generated through an appropriate choice of kernel [118]. The disadvantage of support vector machines is that the classification result is purely dichotomous, and no probability of class membership is given.

#### 2.6.6. Extreme Learning Machine 

Extreme learning machine is the feed forward network [187–189]. It consists of three layers which are similar to the other networks. The only difference is that the hidden elements can be independent from the training data and target functions. Because of this independence of hidden elements, this feed forward network provides better generalization performance and it can learn much faster as compared to the other conventional algorithms.

The important features of extreme learning machine are that even simple math is enough for it. It is a simple tuning-free three-step algorithm. The learning speed is extremely fast. Unlike the traditional classic gradient-based learning algorithms which often face several issues like local minima, improper learning rate, and overfitting. The extreme learning machine tends to reach the solutions straightforward without such trivial issues [190]. This learning algorithm looks much simpler than many other learning algorithms like neural networks and support vector machines.

There is very less work being done on the classification of dermoscopic images using extreme learning machine. Research work done on extreme learning machine shows that extreme learning machine needs much less training time as compared to popular BP and SVM. The prediction accuracy of ELM is usually slightly better than BP [177] and close to SVM in many applications. Compared with BP and SVM, extreme learning machine can be implemented easily since there is no parameter to be tuned except an insensitive parameter *L*. It should be noted that many nonlinear activation functions can be used in extreme learning machine. Extreme learning machine needs more hidden nodes than BP but much less nodes than SVM. This implies that extreme learning machine and BP have much shorter response time to unknown data than SVM. So, this can be a good area to dig in for future research.

### 2.7. Evaluation of Classification Performance

Evaluation of classification results is an important process in the classification procedure. The papers propose that, for skin lesion classification, three different classification tasks should be used as benchmarks: the dichotomous problem for distinguishing common nevi from dysplastic nevi and melanoma, the dichotomous problem for distinguishing melanoma from common nevi and dysplastic nevi, and the trichotomous problem for correctly distinguishing all the three classes.

The two criteria to assess the quality of a classification model are discrimination and calibration. Discrimination is a measure of how well the two classes in the data set are separated and calibration is a measure of how close the predictions of a given model are to the real underlying probability based on expert knowledge. Some of the common measures of analysing discriminatory power of different methods are reported in this paper as can be noticed in Table 3.

Sensitivity and specificity are the most commonly used performance evaluation parameters in the literature. Accuracy can be used as a single parameter, but if there is imbalance between the classes (melanoma, benign), then accuracy is not a suitable approach of evaluation. A better performance measure in unbalanced domains is the receiver operating characteristic (ROC) curve. AUC is a statistically consistent and a more discriminatory measure than accuracy [191, 192]. The log diagnostic odds ratio is also sometimes used in meta-analyses of diagnostic test accuracy studies due to its simplicity (being approximately normally distributed). *d*_class_ is a measure to compare different classifiers presented by Sboner et al. [193] that enable giving a simple estimation of how useful one classifier is with respect to another. By using this parameter instead of accuracy, out the comparison between classifiers can be carried in an accurate but intuitive way, avoiding the unbalanced class problem.

To provide an unbiased estimate of a model's discrimination and calibration there are some important considerations like the effect of class imbalance, train/test ratio, and cross-validation. Several studies have demonstrated that the accuracy degradation on unbalanced data sets is more severe when the classes overlap significantly [190, 194, 195] which is the case in skin lesion classification. Most classifiers focus on learning the large classes which leads to poor classification accuracy for the small classes such as classifying the minority (melanoma) samples as majority (benign) which implies serious consequences.

Train to test ratio is another important factor effecting the classification result. It has been observed [134] that as the training-set size increases, the results improve. The effect of train/test ratios on classification accuracy is studied in [196] and the best classification results were reached with 70/30 train to test ratio. We observed that over training may also lead to less accuracy.

There are two approaches for selecting training and test data: either to separate test and training feature vectors or pick training feature vectors as a subset of the test vectors. A classification result may be overly optimistic if performance cannot be measured on a data set not used for model building. In the ideal case, testing on a separate data set will provide an unbiased estimate of generalization error. If the original data set is too small for this approach, the recommended strategy is to use cross-validation [197] or bootstrapping [198] to make the best possible use of the limited amount of data. One way is to divide the whole data into *n* pieces, *n* − 1 pieces used for training, and the last piece as the test set. This process of *n*-fold cross-validation builds *n* models; the numbers reported are the averages over all *n* test sets. The extreme case of using only one data item for testing is known as leave-one-out cross-validation. Bootstrapping is rarely used in the literature for skin lesion case, but it has shown to be superior to cross-validation on many other data sets [199].

### 2.8. Selection of Suitable Classification Method

The increasing number of electronic data bases containing dermoscopic images has led to an increasing interest in their utilization for building classification models that can “learn” from examples. The need to use data and learning techniques in order to make correct diagnosis requires proper choice of the learning algorithms and of their statistical validation. The problem is difficult given the relative paucity of lesion data and consequently the low quality of training data available and the imbalance between the classes.

**Figure 5:**
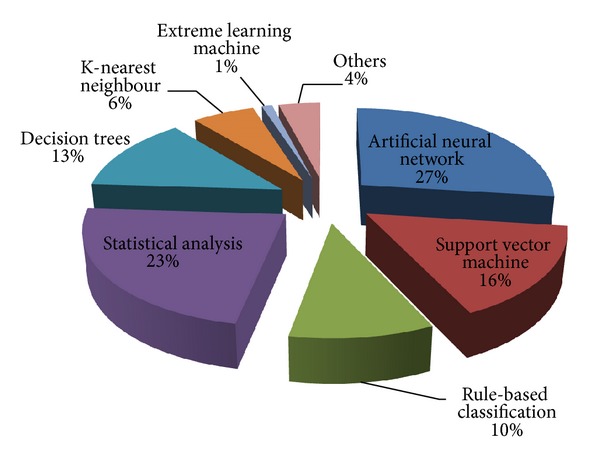
Illustration of classification methods as used by existing diagnostic systems.

A variety of statistical and machine learning approaches are used for the classification of dermoscopic images. As illustrated in Table 4, while Figure 5 presents the percentage of classification methods as used by existing diagnostic systems in literature.

Different classification methods have their own merits. The question of which classification approach is suitable for a specific study is not easy to answer. Different classification results may be obtained depending on the classifier(s) chosen, differences in sample sizes, proportion of melanomas in the sample, and the number of features used for discrimination as can be notice in Table 5. Many factors, such as different sources of obtaining dermoscopic images, availability of classification software, time consumption, computational resources, and the number of melanoma and benign images available for training must be taken into account when selecting a classification method for use.

Very few researchers provided comparisons of different classification algorithms using the same set of images [46, 94, 126, 166, 196]. The review of all these comparative studies reveals that MLP gives better performance than Bayesian and kNN classifiers, while SVM with RBF kernel normally outperforms MLP, decision trees, and other statistical methods. The results of an experimental assessment of the different designs can be the basis for choosing one of the classifiers as a final solution to the problem.

It had been observed in such design studies that although one of the designs would yield the best performance, the sets of patterns misclassified by the different classifiers would not necessarily overlap. These observations motivated the relatively recent interest in combining classifiers. The idea is not to rely on a single decision making scheme. Instead, all the designs, or their subset, are used for decision making by combining their individual opinions to derive a consensus decision. Some classifier combination schemes have been devised [126, 193, 200] for dermoscopic images and it has been experimentally demonstrated that some of them consistently outperform a single best classifier. However, there is presently inadequate understanding why some combination schemes are better than others and in what circumstances.

## 3. Model Validation

A vast number of diagnostic algorithms/models are published each year. Such models do not always work well in practice, so it is widely recommended that they need to be validated [201, 202]. To be useful, a prognostic index should be clinically credible and accurate and have generality (i.e., be validated elsewhere), and the study should be described in adequate detail. To gauge the current state of reporting results in the literature, we sampled many papers on dermoscopic images data sets analysis.

We reviewed 31 publications which claimed fully automatic diagnostic models. We found frequent shortcomings both reporting and methodology used. The paper is proposing some criteria as quality assessment criteria which can be noticed in Table 6. It includes lack of calibration in image acquisition, unspecified method for extracting and selecting variables in the model, and risk of overfitting through too few events per variable. Many researchers did not specify the test/train or used uneven number of melanoma and benign images for training which may lead to biased classification. Some articles do not report comparisons and cross-validation; instead they just reported the performance of a single method. It is imperative that these details should be presented in papers as otherwise the validity of the claims in the papers cannot be assessed by the reader.

When assessing the quality of the results obtained using any diagnostic models, the work should consider the quality of the data set employed in model building, the care with which adjustable model parameters were chosen, and the evaluation criteria used to report the results of the modelling process. This is important in distinguishing between overly optimistic claims (such as when performance is reported on the training set) and needlessly pessimistic ones (when model parameters are chosen in a suboptimal manner). The latter is especially common in studies that promote “new” algorithms.

Apart from all this, in order to judge the performance of an automatic diagnostic model it is important to mention who is going to use that model. If automated diagnostic systems will be used by general practitioners or in pharmacies and shopping centres, these systems should be used with very high sensitivity and reasonably good specificity. That is, it should recognize the greatest number of melanomas in early stage, without misclassifying too many nevi so that unnecessary excision of benign lesions could be avoided.

If the target is the expert user, studies should be designed with the aim to help clinicians in distinguishing between benign lesions, dysplastic nevi, and malignant tumors of the skin. An increase in specificity might be the goal for an automated system directed to expert users together with sensitivity at least equal to that achieved by the expert.

Overall, our objective is to get a classifier with the sensibility and the specificity balanced. It should be noted that the ability to diagnose correctly melanoma is by far the most important property that an automated system must have. The consequence of failure to diagnose correctly a malignant tumor may lead to the eventual death of the patient. On the other hand, if we get a classifier with a high sensibility but a low specificity, it is not going to be useful as a screening method to avoid biopsies (an invasive technique). And, off course, we want a classifier with a high sensibility to avoid false negatives.

## Figures and Tables

**Table 1:** In vivo imaging techniques for the diagnosis of skin cancer.

Method	Advantages	Limitations
Photography [203–205] Total body photograph (2-D TBP 3-D TBP) Baseline photographs of individual lesions	Affordable and easy data management. Monitoring patients with many dysplastic nevi. Useful in the follow-up management and easy comparison for detecting change in size, shape, or color that may be suggestive of malignancy. 3D representation of the patient's entire cutaneous surface may reduce work time and clarify documentation.	Limited morphologic information.

Dermoscopy [8, 10, 19, 123, 206–208] ELM (oil/slide mode and polarizing mode)	Facilitating 20–70% magnification of the skin. Melanoma dermoscopic characteristics are well correlated to histopathologic features. Identifying foci of melanoma for helping pathologist as to where to section the specimen so as to minimize false-negative results as a result of sampling error. Liquid immersion provides increased illumination and resolution and sharper and less distorted colours. Polarizing mode avoids a potential source of nosocomial infections.	Qualitative and potentially subjective. Low magnification in routinely used instruments and the limited scope of observable structures restrict the usefulness and diagnostic applicability of the method.

Multispectral imaging [209–211] Melafind Solar scan Spectrophotometric intracutaneous analysis	Spectral imaging is quantitative and more objective. Less interphysician variability. Melafind can create multispectral sequence of images in less than 3 seconds. SIA scope can help in the diagnosis of lesions as small as 2 mm. Analysing the location, quantity, and distribution of skin chromophores within epidermis and papillary dermis.	Difficult interpretation because of the complexity of the optical processes of scattering and absorption.

Laser-based enhanced diagnosis [212–214] Confocal scanning laser microscopy Reflectance confocal microscopy Spectrally encoded confocal microscopy	In vivo imaging of skin lesions at variable depths in horizontal planes and examination at a quasi-histological resolution without biopsy. High resolution allows imaging of nuclear, cellular, and tissue architecture of the epidermis and underlying dermal structures without a biopsy and allows recognition of abnormal intraepidermal melanocytic proliferation. No tissue damage because of low-power laser.	Processes in the reticular dermis and tumor invasion depth cannot be evaluated reliably. Technically sensitive and expensive to use in routine clinical application. Formal training and experience are required to become proficient in this new technique.

Optical coherence tomography [215–217]	Depth of invasion can be better measured with OCT than CSLM. Noninvasive assessment and monitor of inflammatory skin diseases.	Limited resolution does not allow a differential diagnosis between benign and malignant lesions. Limited to thin tumors because of the strong scattering of epidermic tissue.

Ultrasound imaging [218, 219]	Can provide information about perfusion patterns of lymph nodes and other soft tissues that can be used to stage the tumor.	May overestimate or underestimate tumor thickness; accuracy of results depends heavily on the skill of examiner and anatomic site of lesion.

Magnetic resonance imaging [220–222]	Obtaining information on the depth and extent of the underlying tissue involvement and can be used to measure melanoma thickness or volume.	The need for sufficient resolution and adequate number of images per sequence for discriminating skin lesions.

**Table 2:** Methods for segmentation of dermoscopic images.

Method	Description	Related references
Thresholding	Determining threshold and then the pixels are divided into groups based on that criterion. It include bilevel and multithresholding	Histogram thresholding ([90, 91, 109, 110, 223, 224]) Adaptive thresholding ([61, 88, 111, 112, 124, 126, 200, 225])

Color-based segmentation algorithms	Segmentation based on color discrimination. Include principle component transform/spherical coordinate transform	[134, 226–230]

Discontinuity-based segmentation	Detection of lesion edges using active contours/radial search techniques/zero crossing of Laplacian of Gaussian (LoG)	Active contours ([58, 62, 64, 88, 231–233]) Radial search ([115, 234, 235]) LoG ([117, 163, 236, 237])

Region-based segmentation	Splitting the image into smaller components then merging subimages which are adjacent and similar in some sense. It includes Statistical region merging, multiscale region growing, and morphological flooding	Split and merge ([238, 239]) SRM ([59, 70, 92, 112]) Multi-scale ([118, 196]) Morphological flooding ([79])

Soft computing	Methods involve the classification of pixels using soft computing techniques including neural networks, fuzzy logic, and evolutionary computation	Fuzzy logic ([60, 76, 80, 85, 140, 157, 200, 240]) Neural Network ([177, 241]) Optimization algorithms ([241–243])

**Table 3:** Measures for evaluating performance of a classifier.

Evaluation parameters	
$\text{Accuracy}=\frac{\text{TP}+\text{TN}}{\text{TP}+\text{TN}+\text{FP}+\text{FN}}\times 100\%$	
$\text{Diagnostic accuracy}=\frac{\text{TP}}{\text{TP}+\text{FP}+\text{FN}}$	
$\text{Sensitivity}=\frac{\text{TP}}{\text{TP}+\text{FN}}\times 100\%$	

$\text{Specificity}=\frac{\text{TN}}{\text{TN}+\text{FP}}\times 100\%$	
ROC curve − a plot of the true positive TP-rate versus false positive FP-rate	
$\text{Positive predictive value}=\frac{\text{TP}}{\text{TP}+\text{FP}}\times 100\%$	
$\text{Negative predictive value}=\frac{\text{TN}}{\text{TN}+\text{FN}}\times 100\%$	
$\text{Error probability}=\frac{\text{FP}+\text{FN}}{\text{TP}+\text{TN}+\text{FP}+\text{FN}}\times 100\%$	
Index of suspicion $=\frac{\text{TP}+\text{FP}}{\text{TP}+\text{FN}}\times 100\%$	
${\text{LR}}_{+}=\frac{\text{Sensitivity}}{1-\text{Specificity}}$	
${\text{LR}}_{-}=\frac{1-\text{Sensitivity}}{\text{Specificity}}$	
Diagnostic odds ratio [191], DOR $=\frac{\text{TP}/\text{FN}}{\text{FP}/\text{TN}}$	
Distance of a real classifier from the ideal one $d_{\text{class}}=\sqrt{{\left(1-\text{Se}\right)}^{2}+{\left(1-\text{Sp}\right)}^{2}}$	

**Table 4:** Classification methods used in the literature for skin cancer diagnosis.

Classification method	Related references
*K*-nearest neighbour	[124, 126, 163, 166, 200]

Decision trees	[87, 110, 146, 166] ADWAT [93, 244], Logistic Model tree (LMT) [111, 112, 225], CART [46, 245]

Statistical (discriminant analysis/logistic regression/multifactorial analysis)	[80, 96, 120, 134, 141, 231, 246] DA [117, 121, 247, 248] mathematical classifier [249], logistic regression [38, 166, 236, 237, 239, 250] linear classifier [163]

Rule-based classification	[125, 223, 223, 251, 251–254]

Artificial neural network	[41, 90–92, 94, 126, 135, 140, 141, 157, 166, 177, 196, 209, 230, 246, 248, 254–258]

Support vector machine (SVM)	[40, 46, 61, 77, 91, 94, 118, 166, 196, 200, 230, 259, 260]

Extreme learning machine	[177]

Others (Gaussian maximum likelihood, Bayesian classifier)	[46, 126, 200]

**Table 5:** Summary of classification performance of some skin cancer detection methods.

Source	year	No. of features selected	Classifier	Total images	Melanoma %	Dysplastic nevi %	Benign	Sens %	Spec %	Accuracy
[249]	1993		CART	353	62		38	94	88	
[49]	1994		CART	404	59		41	90	88	80
[121]	1994	22	D.A.	164	11		89	88	89	
[255]	1994		ANN	200	40	30	30	95	88	
[129]	1994	14	ANN	240 216	50 50	16.7	33.3 50	79.5 86	86.3 85.5	82.9 85.7
[261]	1997		Logistic regression	170	44		56	93	67	
[262]	1998	22	Discriminant analysis	917	7		93	93	95	
[257]	1998	16	ANN	120	32.5	48.4	27.5	90	74	
[263]	1999	26	Discriminant analysis	383	4.7		95.3	100	92	
[258]	1999	26	ANN	44	43.2		56.8	97.7	100	
[117]	1999	13	Discriminant analysis	147	38.8		61.2	88	81	85
[45]	2000	38	ANN	315	13.3		86.7	92.9	97.8	
[159]	2001	21	kNN	5363	1.8	18.8	79.4	87	92	
[211]	2001	13	Linear classification	246	25.6	45.1	29.2	100	85	
[41]	2002	13	ANN	588	36.9		63.1			94
[246]	2002	10	ANN	147	38.8		61.2	93	92.8	
[80]	2003	1	Linear classifier	100	50		50	78	90	
[193]	2003	38	LDA + kNN + decision tree	152	27.6		72.4	81	74	
[163]	2004	10	Linear classifier KNN	840	46.5		53.5	95 98	78 79	
[77]	2004		SVM (third degree polynomial)	977	5.12		94.88	96.4	87.16	
[40]	2005	NR	SVM	477	8.8		91.2	84	72	
[248]	2005	20	ANN Discriminant analysis	34	41	59		86 79	100 90	
[259]	2006	200	SVM	22	45		65			70
[87]	2006	28	Decision tree	224	51.8		48.2	51	97	
[237]	2006	3	LR+ multivariate model + ROC	132	17.4		82.6	60.9	95.4	89.4
[118]	2007	18	SVM	564	15.6		84.4	93.3	92.3	
[239]	2007	2	Logistic regression (LR)	260	17.7	18.1	64.2	91.3	81–91	
[200]	2008	10	Multiple classifiers (SVM, GML, kNN)	358	37.4	32.96	29.6			75.69
[124]	2011	33	kNN	83	55.4		44.6	60.7	80.5	66.7
[126]	2011	6	K-NN Bayesian Multilayered perceptron Combination of three	98	52		48	76.4 76.4 78.4 78.4	70.21 85.11 95.74 97.87	73.47 80.61 86.73 87.76
[135]	2012	12	Multilayer percentron	102	50		50	70.5	87.5	76

**Table 6:** Assessment of diagnostic models based on quality assessment criteria.

Criteria	Details Provided (% of models)	Details not Provided (% of models)
Image calibration	51	49
Preprocessing	45	55
Segmentation	78	22
Feature extraction	71	29
Feature selection	54	46
Test/train ratio	42	58
Taking care of balance in lesion classes for training	32	68
Comparative results	55	45
Cross-validation	29	71
